# Academic buoyancy and engagement in EFL learning: a latent growth curve modeling analysis of their dynamic relationship

**DOI:** 10.3389/fpsyg.2026.1944779

**Published:** 2026-10-16

**Authors:** Tengyao Zhang

**Affiliations:** School of Foreign Languages, Shangqiu University, Shangqiu, China

**Keywords:** academic buoyancy, conservation of resources theory, EFL learners, latent growth curve modeling, learning engagement

## Abstract

Grounded in the conservation of resources theory, this 16-week longitudinal study tracked the developmental trajectories of academic buoyancy and learning engagement among college-level English-as-a-foreign-language (EFL) learners in China via repeated questionnaire administrations. Employing latent growth curve modeling (LGCM), this research analyzed changes in both constructs and explored developmental associations between academic buoyancy—a core personal learning resource—and engagement over time. Key findings include: (1) Both academic buoyancy and learning engagement exhibited overall upward trends across the semester, with substantial individual variability observed in initial states and growth rates; (2) Higher latent intercept of academic buoyancy correlated with greater subsequent growth in this construct, whereas the initial state of learning engagement showed no significant association with its own developmental trajectory; (3) The latent intercept of academic buoyancy was positively associated with both the intercept and growth rate of learning engagement, while growth in academic buoyancy aligned with parallel growth in engagement. This study illuminates the dynamic, malleable nature of academic buoyancy and learning engagement in EFL contexts and illustrates the value of LGCM for examining developmental patterns in second language acquisition research.

## 1 Introduction

Against the backdrop of increasing globalization, English continues to serve as a dominant international lingua franca, with its instrumental value remaining central to cross-cultural communication and professional advancement (Köylü and Tracy-Ventura, 2022; Tong and Gao, 2022). Within second language (L2) research, two constructs have emerged as critical predictors of learning outcomes: learners' sustained engagement with academic tasks and their capacity to navigate setbacks (Fu et al., 2026; Xu and Feng, 2024). Academic buoyancy, defined as a context-specific adaptive capacity for managing recurrent minor academic stressors—such as challenging assignments or underperformance on assessments—differs from broader conceptualizations of psychological resilience by focusing on everyday, rather than catastrophic, adversities (Martin and Marsh, 2008). Learning engagement, on the other hand, reflects learners' sustained participation in educational activities at cognitive, emotional, and behavioral levels (Fredricks et al., 2004), functioning as a pivotal indicator for assessing learning quality (Coates, 2005; Tahir and Fatima, 2023). Existing research has established a close association between academic buoyancy and learning engagement in language learning contexts (e.g., Akbaba Eser et al., 2026; Yang and Liang, 2025; Zhai, 2025). However, most studies have relied on cross-sectional designs that can only capture static snapshots of this relationship. These designs also overlook the dynamic nature of language learning and thus fail to explain how academic buoyancy might be correlated with learning engagement over time, especially in the context of ongoing linguistic and cognitive growth.

Conservation of Resources (COR) theory offers a compelling framework for investigating the temporal dynamics between academic buoyancy and learning engagement in English-as-a-foreign-language (EFL) contexts. COR theory is rooted in the premise that individuals are inherently motivated to acquire, retain, and protect valuable resources (Hobfoll, 1989). Academic buoyancy can be conceptualized as a personal resource that enables learners to mitigate resource depletion (Putwain and Nicholson, 2026), potentially helping learners maintain high levels of engagement (Tang et al., 2026). This study aims to longitudinally track the developmental trajectories of academic buoyancy and learning engagement among EFL learners over an academic semester. Through this design, this study examines the initial states, change rates, and predictive relationships between both constructs. This approach addresses key limitations of cross-sectional research, and its findings will provide empirical insights to inform targeted EFL teaching interventions, enhance learners' buoyancy and engagement levels, and advance the understanding of their dynamic processes in L2 learning.

## 2 Literature review

### 2.1 Academic buoyancy

Academic buoyancy, a construct rooted in positive psychology, denotes students' capacity to effectively navigate routine academic adversities encountered in school settings—including underperformance, looming deadlines, examination-related anxiety, and excessive coursework (Martin and Marsh, 2008). Yun et al. (2018) extended this concept to L2 research, noting that daily classroom-based language learning is fraught with fluctuations, stressors, and setbacks, such as high-stakes exam pressure and unsuccessful communicative tasks. Accordingly, they defined language-specific academic buoyancy as learners' aptitude to sustain adaptive motivation, proactively engage with obstacles, and mitigate inherent stressors when confronting these recurrent challenges throughout the L2 learning trajectory (Yun et al., 2018).

Within L2 research, scholarly inquiry primarily centers on two domains: the correlation between academic buoyancy and EFL achievement and its interplay with sociocultural and psychological factors. Concerning the link between academic buoyancy and EFL achievement, cross-cultural research yields consistent findings, demonstrating that buoyancy levels serve as a robust positive predictor of EFL proficiency. For example, Yun et al. (2018) investigated South Korean university students and found that academic buoyancy significantly predicted both English performance and overall grade point average (GPA). They also found that academic buoyancy acted as a mediator between its antecedents (e.g., self-efficacy, teacher-student relationship, and anxiety) and both outcome measures. In a more recent study, Fathi et al. (2025) indicated that a well-developed ideal L2 self and regular mindfulness practices enhanced academic buoyancy, which in turn led to improvements in English achievement among Iranian learners. Similarly, Zhang J. et al. (2026) observed that three dimensions of academic buoyancy—namely, sustainability, positive personal eligibility, and positive acceptance of academic life—played an indirect predictive role in English learning outcomes through the mediating effects of self-/social awareness and self-management competencies.

Regarding the relationship between academic buoyancy and other learner-internal and -external variables, Li et al. (2025) examined the interplay of learning burnout, academic buoyancy, and perceived teacher support among Chinese senior high school EFL learners. Their findings indicated that both perceived teacher support and academic buoyancy directly reduced learning burnout, with teacher support also exerting an indirect mitigating effect via the mediation of academic buoyancy. Extant research further establishes that EFL learners' academic buoyancy is closely intertwined with factors such as learning motivation (Jia and Cheng, 2022), grit, mindfulness, learning boredom (Mohammad Hosseini et al., 2024), learning enjoyment, hope (Gao, 2025), language anxiety, willingness to communicate (Lin et al., 2025), self-efficacy beliefs (Shan, 2026), task-supported instructional approaches (Souzandehfar and Ahmed Abdel-Al Ibrahim, 2023), and classroom social climate (Yang and Liang, 2025).

### 2.2 Learning engagement

Learning engagement describes a state in which learners exhibit heightened attention and active participation throughout the learning process and task completion (Philp and Duchesne, 2016). As both an indicator of effective learning and a desired learning outcome (Liu E. et al., 2024), it serves as a dual marker of instructional success. Learning engagement encompasses agentic, behavioral, emotional, and cognitive dimensions (Fredricks et al., 2004; Reeve and Tseng, 2011). Specifically, agentic engagement involves proactive participation and constructive input in the teaching process (Reeve, 2013). Behavioral engagement denotes observable levels of participation and effort expenditure; emotional engagement centers on affective responses to learning content and activities; cognitive engagement refers to the cognitive efforts exerted to comprehend and master knowledge and skills, including the application of cognitive and metacognitive strategies (Fredricks et al., 2004).

Within the EFL context, learning engagement denotes learners' active commitment to and meaningful involvement in EFL classroom learning activities (Zhong and Zhan, 2024). Language learning engagement has become a focal area of investigation in L2 research. Synthesized research findings reveal that foreign language learning engagement positively impacts both the learning process and its resultant outcomes. Specifically, engagement has been shown to predict students' reading and writing proficiency (Hamedi et al., 2020; Zhu et al., 2023), critical thinking skills (Han et al., 2025), and academic achievements (Cai and Xing, 2025). Researchers have also zoomed in on the factors influencing foreign language learning engagement. Their work has pinpointed learner-external factors—such as perceived teacher autonomy support (Zeng et al., 2026), peer collaboration (Zhang L. et al., 2026), and classroom social climate (Alshewiter et al., 2026)—as pivotal antecedents of engagement among EFL learners. Complementing these external influences, learner-internal factors, such as self-efficacy beliefs (Yang et al., 2026), mindset (Tran and Yough, 2026), academic emotional experiences (Gan et al., 2026; Yang et al., 2026), and learning motivation (Trang, 2026), have also been recognized as critical predictors of EFL learners' engagement.

### 2.3 Relationship between academic buoyancy and engagement in EFL learning

Empirical investigations have consistently demonstrated a positive correlation between academic buoyancy and learning engagement among EFL learners, with this relationship operating through intricate emotional pathways and contextual moderators. Among them, Yang and Liang (2025) utilized structural equation modeling to show that classroom social climate predicted learning engagement via a sequential mediation effect involving academic buoyancy and foreign language learning enjoyment in a sample of Chinese university students. Wang and Liu's (2022) serial mediation analysis further revealed that autonomous motivation influenced learning engagement both directly and indirectly through the chain of academic buoyancy and foreign language boredom, where the standalone mediating effect of academic buoyancy outweighed that of boredom, underscoring its pivotal role in sustaining learning motivation. This positive association is also bounded by specific contextual conditions. For example, Zhao et al. (2025) demonstrated that English-speaking buoyancy exerted a positive influence on speaking engagement directly and indirectly through speaking enjoyment. Besides, speaking anxiety acted as a negative moderator in the buoyancy-engagement relationship. Hu et al.'s (2026) mixed-methods study indicated that EFL learners' perceived teacher support was positively related to their learning engagement, and that academic buoyancy partially mediated this relationship. Liu H. et al. (2024) demonstrated that academic buoyancy could moderate the boredom-engagement relationship, buffering the adverse impact of foreign language boredom on engagement.

Despite significant progress in elucidating the multifaceted connections between academic buoyancy and learning engagement, the reliance on cross-sectional study designs (e.g., Wang and Liu, 2022; Yang and Liang, 2025) constrains the inferences regarding the temporal dynamics between both variables in EFL contexts. For example, while Wang and Liu (2022) proposed the pathway of autonomous motivation → academic buoyancy → boredom → engagement, their cross-sectional design could not confirm the stability of effects over long-term tracking. Therefore, the present study adopts a longitudinal design to examine the relationship between academic buoyancy and learning engagement among college EFL learners and addresses the following research questions (RQs):

RQ1: Over the course of one semester, what are the directional trends and magnitude of change in college students' academic buoyancy and learning engagement in EFL contexts?
RQ2: Do the initial states of academic buoyancy and learning engagement significantly predict their respective rates of change over one semester?
RQ3: What are the developmental associations between the intercept and slope parameters of academic buoyancy and those of learning engagement among college EFL learners?

## 3 Theoretical framework

This study anchors its theoretical framework in the COR theory (Hobfoll, 1989). At its core, the COR theory assumes individuals are inherently driven to acquire, retain, protect, and cultivate resources they perceive as valuable (Hobfoll, 2001). These resources span material assets, social conditions, personal traits, and energy forms (Hobfoll et al., 2018). The theory rests on four interconnected principles. The Primacy of Loss Principle holds that resource loss impacts individuals far more profoundly than resource gain, with even minor losses potentially triggering cascading depletion cycles. The Resource Investment Principle posits that individuals must actively invest diverse resources—including material assets like time and money, personal resources such as self-efficacy and resilience, and conditional resources like social support—to prevent loss, recover from setbacks, or secure new resources. The Gain Paradox Principle highlights that resource gains carry heightened significance during periods of loss, with small gains in stressful contexts yielding greater value than large gains in stable environments. Finally, the Desperation Principle suggests that when resources are exhausted, individuals enter a defensive state to protect themselves, which may manifest in aggressive or irrational behaviors (Hobfoll et al., 2018). Unlike traditional stress assessment theories that prioritize individual perception, COR theory emphasizes the objective value of resources and the role of environmental factors in shaping resource flow, offering a dynamic framework to explain adaptive behaviors under stress.

COR theory provides theoretical grounding for this study's focus on the relationship between academic buoyancy and learning engagement among EFL learners, with its applicability evident across two key dimensions. First, academic buoyancy—defined as the capacity to navigate everyday learning setbacks (Martin and Marsh, 2008)—qualifies as a personal resource within COR theory (Tang et al., 2026). Guided by the Resource Investment Principle, learners with high academic buoyancy are more inclined to invest resources to mitigate loss, recover from challenges, or acquire new assets, thereby fostering sustained learning engagement (Hobfoll et al., 2018). Second, the COR theory frames learning engagement as an outcome of intentional resource investment, with academic buoyancy functioning as a protective mechanism that helps learners minimize resource loss and maintain high engagement levels (Hobfoll et al., 2018).

## 4 Research methodology

### 4.1 Research design

The present study adopts a Latent Growth Curve Model (LGCM) to investigate the dynamic interplay between academic buoyancy and learning engagement among EFL learners. Rooted in regression modeling frameworks, LGCM is a specialized longitudinal analysis technique that simultaneously estimates both individual- and group-level trajectories, enabling researchers to unpack intra-individual change processes and inter-individual differences over extended periods (Hertzog et al., 2008; Lee et al., 2018). To answer the RQs, the present study mainly investigated both group-level trajectories and the associations among the latent growth parameters.

When operationalizing change within the LGCM framework (see Figure 1), it is critical to recognize the model's dual variable structure: measured variables, which consist of repeated measurements of focal constructs—in this case, academic buoyancy and learning engagement—across four time points, and latent variables, which represent unobserved growth parameters that characterize developmental patterns. Within this latent variable structure, two core parameters are typically specified to capture change dynamics: the intercept factor, which represents the initial state of the latent trajectory, and the slope factor, which reflects the rate and direction of change in the construct over the study's duration.

**Figure 1:**
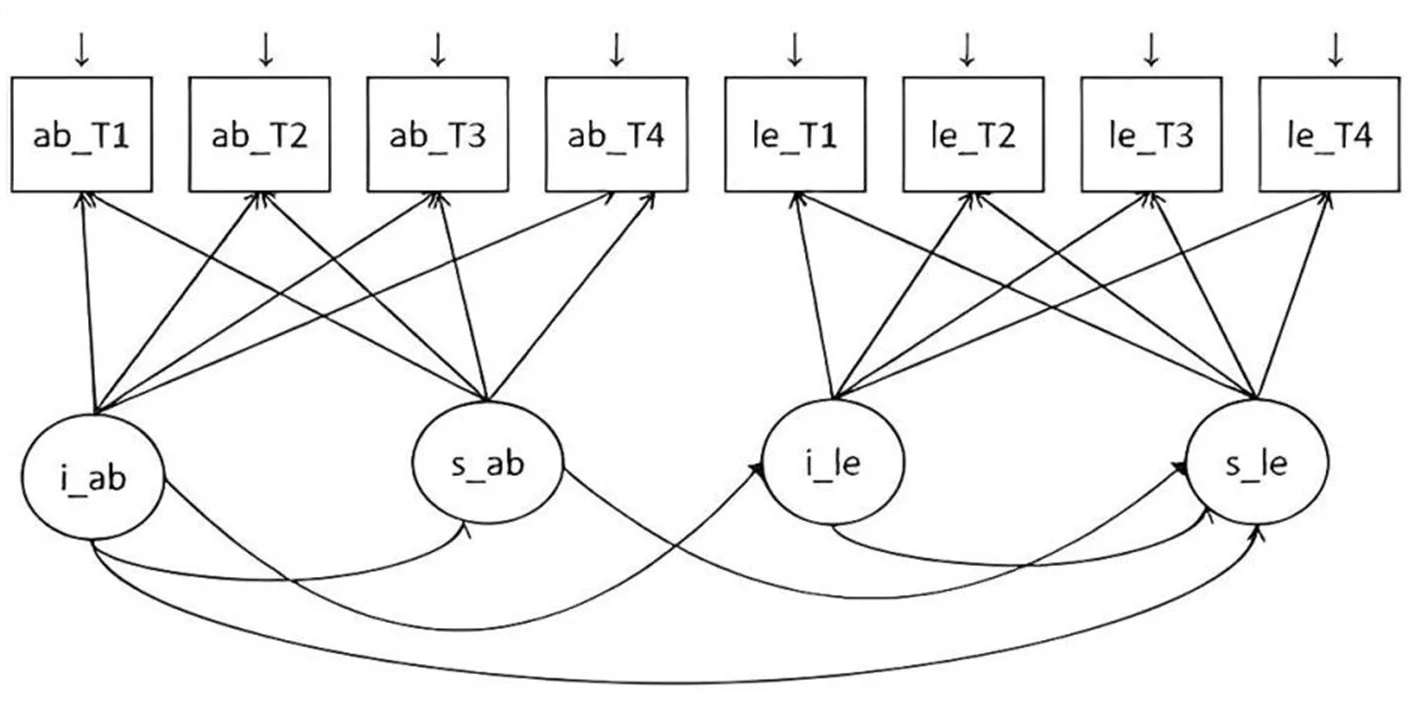
The hypothesized LGCM. Ab, academic buoyancy; le, learning engagement; i, intercept; s, slope.

### 4.2 Participants and procedures

A longitudinal research design was adopted for this study, with convenience sampling implemented to recruit participants while ensuring data integrity. The sample comprised second-year non-English major students from one comprehensive university in Central China. A total of 359 students were initially recruited and completed the first wave (T1) of data collection in early March 2026. Data collection spanned 16 weeks, with follow-up surveys administered at the 6th, 11th, and 16th weeks of the academic semester. Questionnaires were constructed using the *Wenjuanxing* platform, and the survey link was distributed via seven English teachers across 10 intact classes, who forwarded it to their students through class-based social media groups. Unique participant identifiers were assigned to each respondent at T1 to ensure accurate linkage of responses across all four waves. To minimize practice effects associated with repeated survey administration, both the item order and visual layout of questionnaires were systematically varied across each wave of data collection. Prior to survey completion, participants were provided with detailed information about the study's objectives, assured of strict data confidentiality, and informed of their rights to voluntary participation, unconditional withdrawal at any stage without penalty to their course grades, and the requirement to complete an online informed consent form before proceeding.

Of the initial 359 participants enrolled at baseline (T1), 347 completed the T2 assessment (96.7% retention), 339 completed T3 (94.4% retention), and 335 completed the final T4 assessment (93.3% retention). While 335 participants provided data at T4, only 329 participants had complete data across all four waves, indicating that 6 participants exhibited intermittent missingness (i.e., they missed one or more intermediate waves but returned for subsequent assessments). Consequently, the overall attrition rate for the final-wave completion was 6.7% (24/359), whereas the exclusion rate for the complete-case analytic sample was 8.4% (30/359). Among the 329 students participating in all four survey waves, 192 were females and 137 were males, with an age range of 18 to 20 years (*M* = 18.81, *SD* = 0.69). Intraclass correlation coefficients (ICCs) were computed to assess cluster-dependent effects in the data. The resulting ICCs were 0.032 for academic buoyancy, 0.041 for overall learning engagement, and 0.038, 0.045, 0.039, 0.042 for the behavioral, emotional, cognitive, and agentic engagement subdimensions, respectively. While all ICC values fell below the conventional 0.05 threshold (Xu et al., 2025), 329 students were nested within 10 intact classes, with a mean class size of approximately 32.9. Per the standard equal-cluster-size approximation formula *DE*=*1* + (m-1)^*^ICC, where *m* is the average cluster size, calculated design effects ranged from 2.02 to 2.44 across all variables. These estimates confirm that even with only a small share of total variance between classes, class membership still introduced a non-negligible level of dependency into the data. As the 10 classes were distributed across seven teachers, teacher-level dependence could not be fully disentangled from class-level dependence. Given the limited number of higher-level units, a single-level LGCM was used for the primary analysis. To evaluate the robustness of the substantive findings, the final LGCM was re-estimated using class-clustered robust standard errors, with class specified as the clustering variable through the TYPE = COMPLEX option in Mplus. As reported in the Supplementary material, the direction and statistical significance of the focal growth parameters and structural paths were unchanged after this adjustment. Because only 10 classes were available, these results should be interpreted as a sensitivity check rather than as a definitive test of class-level effects.

To examine potential attrition bias, this study compared baseline (T1) demographic characteristics and scores on the primary study variables between the 329 completers and the 30 excluded participants. No significant differences emerged for age (*M* = 18.81, *SD* = 0.69 vs. *M* = 18.73, *SD* = 0.72; *t* = 0.58, *p* = 0.56, *d* = 0.12), gender (χ^2^ = 0.03, *p* = 0.86), baseline academic buoyancy (*M* = 4.17, *SD* = 0.69 vs. *M* = 4.10, *SD* = 0.71; *t* = 0.52, *p* = 0.60, *d* = 0.10), or baseline learning engagement (*M* = 4.24, *SD* = 0.72 vs. *M* = 4.16, *SD* = 0.70; *t* = 0.60, *p* = 0.55, *d* = 0.11). Although these non-significant comparisons could not definitively rule out attrition bias, the low overall attrition rate and the negligible effect sizes suggest that such bias was likely minimal and unlikely to materially affect the substantive conclusions.

### 4.3 Instruments

A composite online questionnaire served as the primary data collection instrument for this study, encompassing four distinct sections. Opening with a concise research introduction, the questionnaire proceeded to gather demographic information, including respondents' gender and age. The final two sections comprised validated Likert-type scales designed to assess two core constructs among EFL learners: academic buoyancy and learning engagement.

Academic buoyancy was measured using a 4-item scale adapted by Yun et al. (2018) from the original scale developed by Martin and Marsh (2008). Example items included statements such as, “I remain motivated even during extended English learning activities spanning several months.” Participants responded to the scale using a 7-point Likert format, where responses ranged from 1 (Strongly Disagree) to 7 (Strongly Agree). A composite academic buoyancy score was computed by averaging responses across the four items in the scale.

Learning engagement was assessed via a 22-item scale modified from Reeve and Tseng's (2011) *Learning Engagement Scale*, which operationalizes engagement across four dimensions: agentic engagement (5 items), behavioral engagement (5 items), cognitive engagement (8 items), and emotional engagement (4 items). To enhance contextual relevance for EFL learners, minor semantic adaptations were made by replacing general references to “class,” “teacher,” and “schoolwork” with domain-specific terms: “English class,” “English teacher,” and “English coursework.” Representative items for each dimension included: “During English class, I ask questions” (agentic), “I listen carefully in English class” (behavioral), “When doing English coursework, I try to relate what I'm learning to what I already know” (cognitive), and “I enjoy learning new things in English class” (emotional). This scale employs a 7-point Likert response format (1 = “Strongly Disagree,” 7 = “Strongly Agree”). All items are positively worded, and scores were computed by first averaging responses within each of the four subscales, then calculating an overall mean across subscales to generate a global engagement mean score.

To mitigate participants' cognitive load, a rigorous back-translation procedure was employed to ensure cross-linguistic equivalence when adapting the original English scales into Chinese. This process began with two independent bilingual translators with applied linguistics backgrounds generating separate Chinese translations of the English scales. These initial versions were then compared and synthesized by the researcher to produce a unified Chinese draft. Next, two English-language specialists who had not been exposed to the original English scales performed blind back-translations of the Chinese version into English. The researcher then compared these back-translations against the original scales to verify semantic consistency, resolving any discrepancies through iterative discussion and refinement. Finally, three educational psychology experts evaluated the Chinese scales for content validity, cultural appropriateness, and linguistic clarity, providing targeted revisions that were integrated into the final Chinese instruments. To further ensure interpretive validity, a pilot group of 30 students (15 male, 15 female) reviewed the Chinese questionnaire to assess comprehensibility and readability, confirming its suitability for large-scale data collection.

### 4.4 Data analysis

Data analysis proceeded in three sequential phases. First, descriptive statistical analyses were conducted to characterize the central tendency and variability of academic buoyancy and learning engagement scores across all four measurement waves. Second, longitudinal measurement invariance testing was performed on both scales to confirm that the underlying factor structures of academic buoyancy and learning engagement remained consistent across the study's duration, ensuring comparability of scores across four time points. Finally, LGCM was implemented using Mplus 8.6 software to examine developmental trajectories and interconstruct relationships. To address RQ1, an unconditional LGCM was specified to estimate the intercepts and growth slopes of both academic buoyancy and learning engagement over one semester. For RQ2, the unconditional models were extended by incorporating the intercepts of academic buoyancy and learning engagement as time-invariant predictors into their respective growth models. This analytical framework enabled an examination of whether students' initial states of the latent trajectory in one construct systematically predicted their rate of improvement or decline in the same construct throughout the semester. To answer RQ3, a conditional LGCM was constructed by integrating both the intercept and slope of academic buoyancy into the growth model of learning engagement. This approach allowed for a rigorous test of whether the temporal dynamics of academic buoyancy development, including both initial status and the rate of change, statistically accounted for the developmental trajectory of learning engagement among EFL learners. Models were estimated using the robust maximum likelihood (MLR) estimator, and model fit was evaluated using conventional indices, including CFI > 0.95, TLI > 0.95, RMSEA < 0.06, and SRMR < 0.08 (Hu and Bentler, 1999).

## 5 Results

### 5.1 Descriptive statistics, reliability, and validity tests

Descriptive statistics reported in Table 1 reveal the temporal patterns of academic buoyancy and learning engagement across four measurement points. Academic buoyancy scores showed a generally upward trajectory, with mean values of 4.17 (*SD* = 0.69) at T1, 4.44 (*SD* = 0.71) at T2, 4.22 (*SD* = 0.70) at T3, and 4.63 (*SD* = 0.68) at T4. Similarly, learning engagement exhibited a comparable upward trend, with mean scores of 4.24 (*SD* = 0.72) at T1, 4.43 (*SD* = 0.75) at T2, 4.18 (*SD* = 0.74) at T3, and 4.46 (*SD* = 0.73) at T4. At T3, both constructs showed a temporary decline from T2, with learning engagement dropping below its baseline level, before rebounding at T4. All univariate skewness and kurtosis values fell within the ±2 threshold, confirming the approximate normality of the data distribution (Kline, 2023) and satisfying the univariate normality assumption. However, Mardia's test of multivariate kurtosis indicated a mild departure from multivariate normality across the four waves of data collection. Analyses were conducted on the full set of items measuring academic buoyancy and learning engagement at each time point. The resulting normalized multivariate kurtosis estimates were 4.48, 4.42, 4.51, and 4.45 (all *p*s < 0.05). These values indicated departure from multivariate normality at all four waves. Accordingly, MLR estimation with scaled statistics in subsequent analyses was employed to adjust for non-normality bias.

**Table 1:** Descriptive results.

Variables	Values	T1	T2	T3	T4
Academic buoyancy	Mean (*SD*)	4.17 (0.69)	4.44 (0.71)	4.22 (0.70)	4.63 (0.68)
	Skewness	−0.32	−0.29	−0.33	−0.31
	Kurtosis	0.15	0.11	0.13	0.10
Learning engagement	Mean (*SD*)	4.24 (0.72)	4.43 (0.75)	4.18 (0.74)	4.46 (0.73)
	Skewness	−0.25	−0.23	−0.26	−0.24
	Kurtosis	0.08	0.06	0.09	0.07

Reliability analyses yielded Cronbach's α coefficients of 0.829, 0.803, 0.791, and 0.787 for the academic buoyancy scale from T1 to T4, respectively. For the learning engagement scale, α values were 0.817, 0.824, 0.807, and 0.834 across the four measurement waves. All coefficients surpassed the 0.70 threshold for acceptable internal consistency (Peterson and Kim, 2013), confirming that both scales produced stable and reliable measurements across the semester.

Confirmatory factor analysis (CFA) at T1 supported the one-factor structure of the academic buoyancy scale (χ^2^*/df* = 2.174, CFI = 0.961, TLI = 0.975, RMSEA = 0.043, SRMR = 0.057). A higher-order CFA at T1 was performed to validate the hierarchical structure of learning engagement, framing agentic, behavioral, cognitive, and emotional engagement as first-order factors loading onto a single second-order learning engagement factor. This model yielded acceptable fit indices (χ^2^/*df* = 2.266, CFI = 0.977, TLI = 0.969, RMSEA = 0.052, SRMR = 0.048), with statistically significant factor loadings ranging from 0.72 to 0.80 (*p* < 0.001). Convergent validity of the academic buoyancy scale was established through average variance extracted (AVE) and composite reliability (CR), with an AVE value of 0.63 and a CR coefficient of 0.88, both exceeding the recommended thresholds of 0.50 and 0.70, respectively (Fornell and Larcker, 1981; Peterson and Kim, 2013). A CR value of 0.84 for the higher-order engagement construct confirmed satisfactory internal consistency across its four dimensions (Raykov and Marcoulides, 2011). Discriminant validity was assessed via two methods. First, Heterotrait-Monotrait (HTMT) ratios calculated for the four engagement dimensions all fell below the 0.85 threshold (agentic-behavioral = 0.72, agentic-cognitive = 0.68, agentic-emotional = 0.75, behavioral-cognitive = 0.79, behavioral-emotional = 0.76, cognitive-emotional = 0.73), indicating acceptable discriminant validity (Henseler et al., 2015). Second, a chi-square difference test was performed by comparing a restricted model (correlation between higher-order engagement and academic buoyancy fixed at 1.0) with an unrestricted model (correlation freely estimated). Because estimation used the MLR estimator, the nested-model comparison was carried out with the Satorra-Bentler scaled chi-square difference test rather than the raw difference between two scaled statistics. The corrected difference was significant (scaled Δχ^2^ = 12.34, *df* = 1, *p* < 0.001; Bagozzi and Phillips, 1982), confirming that learning engagement and academic buoyancy represent distinct constructs.

To verify that the scales could yield comparable results across time points, this study tested for measurement invariance, examining configural, metric, and scalar invariance for both the academic buoyancy and learning engagement scales. For the learning engagement scale, measurement invariance was tested in accordance with the hierarchical structure of the construct, with distinct parameters constrained at each stage for both the first-order and the second-order levels. At the configural stage, no parameters were constrained; the same pattern of factor loadings was specified across the four waves, with all first-order factor loadings (items loading onto agentic, behavioral, cognitive, and emotional engagement), second-order factor loadings (the four first-order factors loading onto the higher-order learning engagement factor), item intercepts, and residual variances freely estimated in every wave. At the metric stage, only the loadings were constrained to be equal across waves—specifically, all first-order factor loadings (item → first-order factor) and all second-order factor loadings (first-order factor → higher-order engagement factor) were fixed to be invariant across T1–T4, while item intercepts and residual variances remained freely estimated. At the scalar stage, equality constraints were added to item intercepts, on top of the first- and second-order factor loadings that had already been constrained during the metric invariance stage. This test supported scalar invariance at the observed-indicator level. Because first-order factor intercepts were not constrained across waves, the results did not establish scalar invariance for every level of the complete second-order structure. Across all stages, the second-order factor loadings were constrained in parallel with the first-order loadings, and the residual variances of the first-order factors were freely estimated, as is standard practice for testing invariance in hierarchical models. Results for the learning engagement scale revealed acceptable fit across all invariance models: configural invariance (χ^2^*/df* = 2.232, CFI = 0.969, TLI = 0.968, RMSEA = 0.053, SRMR = 0.041), metric invariance (χ^2^*/df* = 2.359, CFI = 0.953, TLI = 0.966, RMSEA = 0.053, SRMR = 0.058), and scalar invariance (χ^2^*/df* = 2.482, CFI = 0.951, TLI = 0.963, RMSEA = 0.050, SRMR = 0.054). For the academic buoyancy scale, all nested models showed satisfactory fit: configural invariance (χ^2^*/df* = 2.181, CFI = 0.982, TLI = 0.973, RMSEA = 0.055, SRMR = 0.051), metric invariance (χ^2^*/df* = 2.126, CFI = 0.971, TLI = 0.956, RMSEA = 0.046, SRMR = 0.057), and scalar invariance (χ^2^*/df* = 2.352, CFI = 0.953, TLI = 0.952, RMSEA = 0.052, SRMR = 0.056). For comparisons between nested models, changes in CFI, RMSEA, and SRMR were computed for each adjacent pair of sequentially constrained models. Results for the academic buoyancy scale were as follows: between the configural invariance and metric invariance models, ΔCFI = −0.011, ΔRMSEA = −0.009, ΔSRMR = 0.006; between the metric invariance and scalar invariance models, ΔCFI = −0.018, ΔRMSEA = 0.006, ΔSRMR = −0.001. For the learning engagement scale, the same pairwise comparisons produced these results: between configural and metric invariance, ΔCFI = −0.016, ΔRMSEA = 0.000, ΔSRMR = 0.017; between metric and scalar invariance, ΔCFI = −0.002, ΔRMSEA = −0.003, ΔSRMR = −0.004. Following Chen (2007), a lack of invariance is indicated when ΔCFI ≥ 0.01 (a decrease of at least 0.01), accompanied by either ΔRMSEA ≥ 0.015 or ΔSRMR ≥ 0.03 for loadings (and ΔSRMR ≥ 0.01 for intercepts). Applying these benchmarks, certain CFI deviations exceeded the 0.01 cutoff, yet RMSEA and SRMR changes remained within permissible bounds. Considering the changes of all fit indices comprehensively, it is still possible to maintain acceptable measurement invariance across time points.

### 5.2 Direction of change in academic buoyancy and learning engagement

Combined analysis of intercepts, slopes, and covariance structures further confirmed robust model fit for examining developmental trajectories across both constructs (χ^2^*/df* = 2.332, CFI = 0.969, TLI = 0.962, RMSEA = 0.057, SRMR = 0.043). The final model is presented in Figure 2. For the linear slope specification, time scores were coded as [0, 1, 2, 3] corresponding to measurement waves T1 through T4, reflecting equal temporal intervals between assessments. Unconstrained estimation was applied to both intercepts and slopes across academic buoyancy and learning engagement constructs. To enhance model parsimony, residual variances of observed indicators were constrained to equivalence across all time points. Concurrent correlations between residuals of academic buoyancy and learning engagement were freely estimated at each wave to account for shared measurement error within time points (T1: *r* = 0.33, *p* < 0.001; T2: *r* = 0.29, *p* < 0.001; T3: *r* = 0.30, *p* < 0.001; T4: *r* = 0.28, *p* < 0.001).

**Figure 2:**
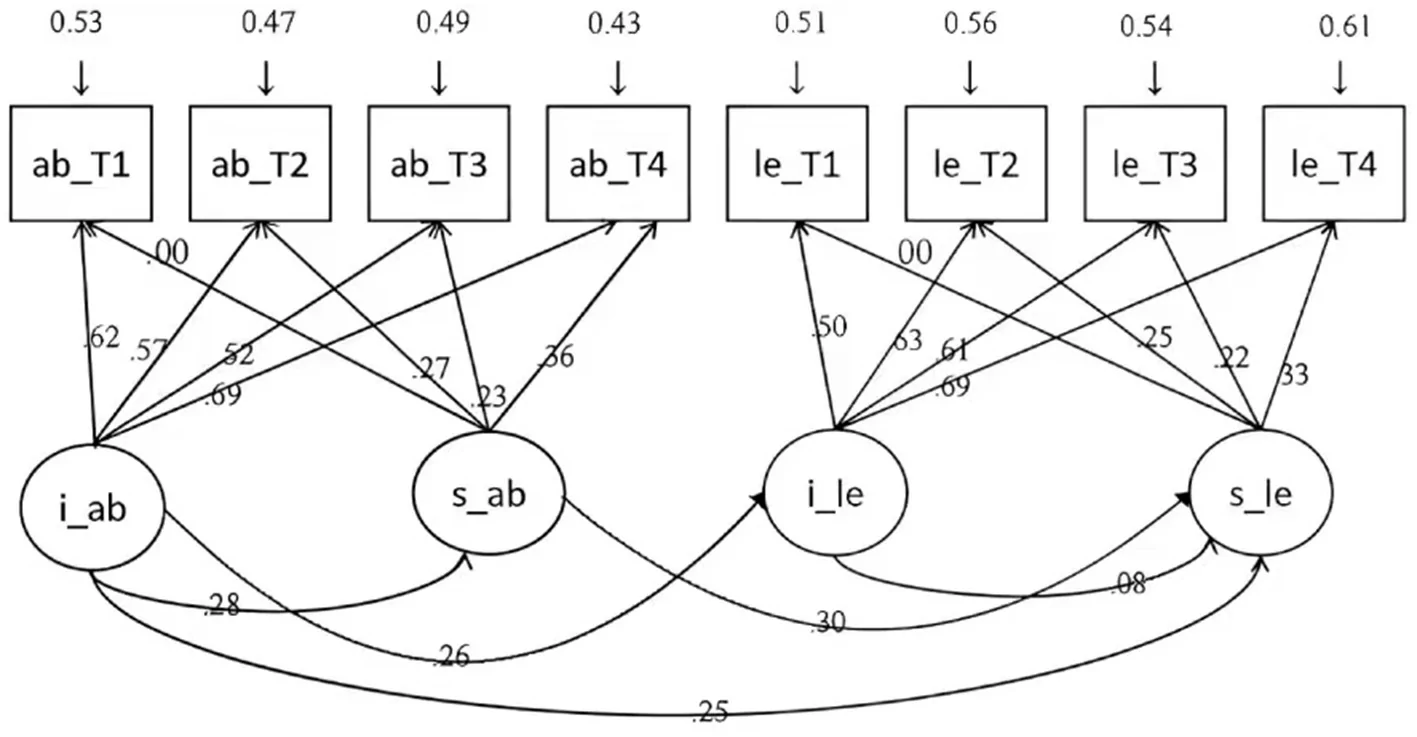
The final LGCM.

To validate the appropriateness of the linear growth specification, model comparisons were conducted between the linear LGCM, a quadratic growth model, and an unstructured non-linear model. The quadratic growth model was specified with an intercept factor, a linear slope factor (time scores coded as [0, 1, 2, 3]), and a quadratic factor (time loadings of [0, 1, 4, 9]). The quadratic factor means for academic buoyancy and learning engagement were freely estimated, whereas their variances and covariances with the intercept and the linear slope were fixed to zero. Under this parameterization, two additional free parameters were introduced relative to the linear model, so that the quadratic model differed from the linear model by two degrees of freedom. The unstructured nonlinear model was parameterized by fixing the loading of the first time point (T1) to 0 and that of the second time point (T2) to 1, with the T3 and T4 loadings freely estimated and constrained to be equal across the two growth processes so as to capture the shared nonlinear trajectory. This specification likewise added two free parameters relative to the linear model. Corresponding fit indices and parameter estimates are reported in Table 2. The quadratic model produced non-significant quadratic terms for both academic buoyancy (*b* = −0.012, *SE* = 0.015, *p* = 0.421) and learning engagement (*b* = −0.008, *SE* = 0.013, *p* = 0.537), indicating no meaningful curvilinear trajectory patterns. As shown in Table 2, the unstructured model failed to demonstrate substantive fit improvement while exhibiting higher AIC and BIC values, consistent with model overfitting. Based on these results, the linear model was retained as the most parsimonious specification with robust empirical support. Model comparisons for nested models were conducted using the Satorra-Bentler scaled chi-square difference test, as recommended for MLR estimation. In both comparisons, the non-significant difference tests (Δχ^2^ = 1.8, *df* = 2, *p* = 0.407 for the quadratic model; Δχ^2^ = 3.1, *df* = 2, *p* = 0.212 for the non-linear model) supported the retention of the more parsimonious linear specification.

**Table 2:** Comparison of linear and non-linear LGCM models.

Model	*χ^2^/df*	CFI	TLI	RMSEA	SRMR	AIC	BIC	*Δχ*^2^ (*df*, *p*) vs. linear
Linear	2.332	0.969	0.962	0.057	0.043	1568.2	1624.5	–
Quadratic	2.289	0.971	0.965	0.056	0.042	1570.5	1638.8	1.8 (2, 0.407)
Non-linear	2.256	0.972	0.967	0.055	0.041	1572.1	1650.3	3.1 (2, 0.212)

Table 3 presents the principal LGCM coefficients. With respect to academic buoyancy, the estimated mean intercept was 4.171 (*SE* = 0.075, *p* < 0.001). The mean scores of both constructs underwent a slight decrease at T3; however, the mean slope parameter of academic buoyancy was 0.124 (*SE* = 0.019, *p* < 0.001), demonstrating a general upward trend over time, with an average change of 0.124 per measurement occasion. Regarding learning engagement, the mean intercept was estimated at 4.239 (*SE* = 0.069, *p* < 0.001), accompanied by a mean slope of 0.051 (*SE* = 0.022, *p* = 0.022), also indicating a general upward trajectory across the data collection period, with an average change of 0.051 per assessment point.

**Table 3:** Principal LGCM coefficients for academic buoyancy and learning engagement.

Constructs	Parameter	Unstandardized Estimate (*SE*)	95% C.I.	*p*-value
Academic buoyancy	Intercept	4.171 (0.075)	[4.024, 4.318]	< 0.001
	Slope	0.124 (0.019)	[0.087, 0.161]	< 0.001
	Intercept variance	0.678 (0.093)	[0.496, 0.860]	< 0.001
	Slope variance	0.033 (0.007)	[0.019, 0.047]	< 0.001
Learning engagement	Intercept	4.239 (0.069)	[4.104, 4.374]	< 0.001
	Slope	0.051 (0.022)	[0.008, 0.094]	0.022
	Intercept variance	0.391 (0.150)	[0.097, 0.685]	0.009
	Slope variance	0.044 (0.014)	[0.017, 0.071]	0.002

Variance components revealed significant individual differences in both initial states and growth rates. For academic buoyancy, significant variance was observed in initial scores (σ^2^ = 0.678, *SE* = 0.093, *p* < 0.001) and growth slopes (σ^2^ = 0.033, *SE* = 0.007, *p* < 0.001). Parallel findings emerged for learning engagement, with significant variance in initial levels (σ^2^ = 0.391, *SE* = 0.15, *p* =.009 < 0.05) and growth trajectories (σ^2^ = 0.044, *SE* = 0.014, *p* = 0.002 < 0.05). These results confirm substantial heterogeneity in participants' starting levels of academic buoyancy and learning engagement, as well as distinct patterns of growth over the course of the semester.

### 5.3 Predictive effects of both constructs' initial states on their rates of change

As shown in Figure 2 and Table 4, the intercept of academic buoyancy emerged as a significant positive predictor of its subsequent growth rate (*b* = 0.062, β = 0.28, *p* = 0.001, 95% CI [0.024, 0.099]), indicating that students with higher initial states of academic buoyancy exhibited more rapid improvements in this construct over the course of the semester. In contrast, the intercept of learning engagement did not significantly predict its rate of change (*b* = 0.027, β = 0.08, *p* > 0.05, 95% CI [−0.036, 0.090]), suggesting that students' initial levels of engagement were not associated with the pace at which their engagement developed throughout the study period.

**Table 4:** LGCM latent variable path coefficients.

Paths	Unstandardized coefficient (*SE*)	β	95% C.I.	*p*-value
ab_intercept → ab_slope	0.062 (0.019)	0.28	[0.024, 0.099]	0.001
le_intercept → le_slope	0.027 (0.032)	0.08	[−0.036, 0.090]	0.395
ab_intercept → le_intercept	0.197 (0.057)	0.26	[0.085, 0.310]	< 0.001
ab_intercept → le_slope	0.064 (0.024)	0.25	[0.017, 0.111]	< 0.01
ab_slope → le_slope	0.346 (0.098)	0.30	[0.155, 0.538]	< 0.001

### 5.4 Predictive effect of academic buoyancy on learning engagement

The intercept and slope of academic buoyancy were incorporated as predictors into the growth model of learning engagement. Results in Figure 2 and Table 4 reveal that the intercept of academic buoyancy significantly positively predicted that of learning engagement (*b* = 0.197, β = 0.26, *p* < 0.001, 95% CI [0.085, 0.310]), indicating that students with higher initial states of academic buoyancy exhibited correspondingly higher initial states of learning engagement. Additionally, the intercept of academic buoyancy significantly positively predicted the rate of change in learning engagement (*b* = 0.064, β = 0.25, *p* < 0.01, 95% CI [0.017, 0.111]), suggesting that students with higher starting academic buoyancy demonstrated more rapid growth in learning engagement over time. Finally, the rate of change in academic buoyancy significantly positively predicted the rate of change in learning engagement (*b* = 0.346, β = 0.30, *p* < 0.001, 95% CI [0.155, 0.538]), indicating that students who exhibited faster improvements in academic buoyancy also experienced accelerated growth in learning engagement, demonstrating a synchronous developmental trajectory between both constructs.

## 6 Discussion

This longitudinal study examined the dynamic developmental associations between EFL learners' academic buoyancy and learning engagement by tracking their trajectories over one semester via LGCM. Key findings reveal that both constructs exhibited overall upward trends. From the perspective of COR theory (Hobfoll, 1989), the overall growth pattern of academic buoyancy (i.e., the ability to navigate daily academic challenges in EFL learning in the present study) could be seen as a gradual expansion of personal resources. As a pivotal personal resource, learners' higher initial status of academic buoyancy could be interpreted via COR theory as allowing for more strategic resource investment (Smith, 2020), which may potentially accelerate self-improvement over the semester. Concurrently, gradual enhancement of learning engagement mirrored developmental patterns observed in academic buoyancy. A possible account, consistent with COR theory, is that if students' personal resource reservoirs expand, their active engagement and effortful involvement may correspondingly deepen (Yang and Liang, 2025). In contrast to the growth trajectory observed for learning engagement, the increase in academic buoyancy was larger in magnitude. On the original 1–7 scale, the model implied an average increase of 0.124 points per wave for academic buoyancy (*SE* = 0.019) and 0.051 points per wave for learning engagement (*SE* = 0.022); the wide slope variance indicates considerable heterogeneity around these averages. The growth in learning engagement, while smaller in magnitude, reflects a general developmental pattern that should not be overlooked due to its close association with long-term language acquisition (Xu et al., 2026). Notably, both constructs remained at moderate levels across the semester, with a slight dip at T3 signaling the inherent variability and non-linearity in language learning processes (Toffoli, 2020). The subsequent rebound at T4 observed in group-level data confirms this fluctuation was transient and did not disrupt the overall upward aggregate trajectory. However, the LGCM further revealed significant individual differences in both initial states and growth rates, as evidenced by the significant slope variance for both constructs. This highlights critical inter-individual differences in developmental patterns, underscoring the need for personalized instructional approaches.

The study also shows that the latent intercept of academic buoyancy emerged as a robust positive predictor of its own subsequent change within the LGCM framework, underscoring the critical role of initial resource endowments. According to COR theory's resource investment principle (Hobfoll et al., 2018), students with higher academic buoyancy may be understood as better positioned to protect and build upon their personal resources. When confronted with routine academic adversities, their response patterns could plausibly reflect adaptive coping that supports resource maintenance (Skinner and Pitzer, 2012). In contrast, the latent intercept of learning engagement showed no significant statistical association with its own growth rate. This null finding may stem from the nature of learning engagement itself. Unlike more stable individual traits, learning engagement is a multidimensional and dynamically evolving state (Wang and Degol, 2014). Learners' engagement levels can be influenced not only by individual resources but also by contextual elements such as instructional scaffolding and task complexity (Qiu, 2026; Saracostti et al., 2024) and psychological variables such as motivational orientation and affective experiences (Kara et al., 2024; Purarjomandlangrudi and Chen, 2020). Consequently, a singular baseline measure may not independently account for the long-term developmental trajectory of learning engagement.

Finally, the cross-dimensional statistical associations between academic buoyancy and learning engagement—encompassing links between latent intercepts and growth trajectories—provide empirical support for applying COR theory as an interpretive framework in educational contexts. Students with higher initial academic buoyancy parameters not only exhibited greater initial learning engagement but also demonstrated steeper growth trajectories over time within the LGCM model. This pattern observed at the group level suggests that academic buoyancy, as a core personal resource (Tang et al., 2026), tends to develop in tandem with holistic engagement growth (Qian and Qiu, 2026). Vîrgǎ et al. (2022) observed similar patterns, finding that psychological capital—comprising resilience, self-efficacy, and other personal resources—shows positive associations with academic performance through learning engagement pathways. The findings of the current study extend cross-sectional research indicating positive links between EFL learners' academic buoyancy and learning engagement (e.g., Hu et al., 2026; Zhao et al., 2025). Crucially, positive statistical associations between changes in academic buoyancy and corresponding changes in learning engagement underscore temporal patterns of resource-engagement dynamics within the specified model. Enhanced academic buoyancy parameters show positive alignment with sustained learning engagement trajectories (Liu et al., 2026; Qian and Qiu, 2026). This longitudinal evidence addresses limitations of prior cross-sectional studies, which often failed to capture temporal developmental patterns, and offers insights into statistical associations between academic buoyancy and learning engagement within the LGCM framework.

## 7 Implications, limitations, and suggestions

This study yields several insightful contributions to educational psychology research. Theoretically, it extends the application scope of the COR theory to EFL learning contexts, clarifies the dynamic predictive relationship between academic buoyancy and learning engagement, and offers a novel lens for analyzing EFL learners' iterative learning processes. Methodologically, this study employed LGCM to characterize the developmental trajectories of academic buoyancy and learning engagement, along with the predictive effects of their initial levels on subsequent change patterns. This approach provides a replicable framework for implementing longitudinal research designs in EFL education. Practically, the findings of this study also carry implications for EFL teaching and learning support. Given that the initial state of academic buoyancy predicts subsequent growth in both academic buoyancy itself and learning engagement, EFL teachers can prioritize early screening of learners' academic buoyancy levels and provide targeted skill-building support for learners with lower initial states of academic buoyancy. The transient dip observed at the mid-semester T3 measurement also suggests that teachers can provide timely scaffolding to help learners maintain their buoyancy and engagement before larger declines occur. The finding of significant individual differences in growth trajectories also indicates that one-size-fits-all intervention strategies may be less effective than adaptive approaches that adjust support intensity based on individual learner needs.

Despite these implications, this study has several limitations that warrant future consideration. First, the sample was restricted to undergraduate students from one selected Chinese university, limiting generalizability. Future research should expand sampling to include diverse regional contexts, institutional tiers, and graduate student populations to enhance external validity. Second, while the 7-point Likert scales used demonstrated acceptable psychometric properties, self-report measures may introduce common-method variance and inherently limit the depth of insight into complex psychological constructs. Integrating qualitative methods, such as semi-structured interviews, classroom observations, and learning analytics data, could provide a more holistic understanding of academic buoyancy and learning engagement dynamics. Third, the study focused solely on the direct relationship between academic buoyancy and learning engagement; future research should investigate mediating factors (e.g., learning motivation, self-efficacy) and moderating variables (e.g., classroom environment, teacher support) to unpack the underlying mechanisms. Fourth, this study primarily examined group-level developmental trends without investigating individual differences in trajectory patterns. Future research could address this limitation by exploring heterogeneous subgroups within the sample. Fifth, the one-semester research period may not capture long-term developmental patterns and cannot definitively establish causal directionality between academic buoyancy and learning engagement. Extending the longitudinal timeframe could reveal more nuanced trajectories and dynamic relationships over extended learning periods. Future research could also employ experimental designs or cross-lagged panel models to clarify temporal ordering. Sixth, the study assumes linear developmental trajectories, which may not fully capture the non-linear dynamics of academic buoyancy and engagement. Future research could explore piecewise growth models or latent class growth analysis to identify heterogeneous trajectory patterns. Additionally, although the class-clustered sensitivity analysis broadly supported the primary findings, the limited number of higher-level units constrained the assessment of contextual dependence. Because the 10 classes were taught by seven teachers, class- and teacher-level influences could not be separately estimated. Future research should recruit a larger number of classes and teachers and apply multilevel models to partition variance across student, class, and teacher levels. Finally, despite an overall low attrition rate, complete-case analysis may still introduce selection bias by excluding participants with missing data. Future research could utilize multiple imputation or full-information maximum likelihood estimation to retain more data and reduce potential bias.

## 8 Conclusion

This study unpacks the dynamic developmental patterns and statistical associations linking academic buoyancy and learning engagement among undergraduate students. These findings offer a nuanced lens for understanding resource management processes and learning dynamics in EFL contexts. Results indicate that academic buoyancy, as a core personal resource, not only exhibits potential for sustained growth over time but also shows positive developmental associations with holistic enhancements in learning engagement. This study extends COR theory to L2 contexts and also provides actionable pathways for fostering student buoyancy and enhancing learning engagement in EFL pedagogical practice. Future researchers can conduct deeper exploration into the micro-level mechanisms underlying the academic buoyancy-engagement relationship, laying the groundwork for more targeted, evidence-based educational interventions.

## Data Availability

The raw data supporting the conclusions of this article will be made available by the authors, without undue reservation.
