# Supplementary material for: Academic buoyancy and engagement in EFL learning: a latent growth curve modeling analysis of their dynamic relationship
Source: Front Psychol. 2026 Oct 16;17:1944779. doi: 10.3389/fpsyg.2026.1944779 (PMC13750305; doi:10.3389/fpsyg.2026.1944779)
Supplement: Supplementary file 1 [file Data_Sheet_1.pdf]

Table S1. LGCM coefficients based on cluster-robust standard errors (Sensitivity Analysis).

**Constructs**	**Parameter**	**Unstandardized Estimate (*SE*)**	**95% C.I.**	***p*-value**
Academic buoyancy	Intercept	4.171 (0.079)	[4.016, 4.326]	< 0.001
Academic buoyancy	Slope	0.124 (0.021)	[0.083, 0.165]	< 0.001
Academic buoyancy	Intercept variance	0.678 (0.098)	[0.486, 0.870]	< 0.001
Academic buoyancy	Slope variance	0.033 (0.008)	[0.017, 0.049]	< 0.001
Learning engagement	Intercept	4.239 (0.073)	[4.096, 4.382]	< 0.001
Learning engagement	Slope	0.051 (0.024)	[0.004, 0.098]	0.034
Learning engagement	Intercept variance	0.391 (0.158)	[0.081, 0.701]	0.013
Learning engagement	Slope variance	0.044 (0.015)	[0.015, 0.073]	0.003

Compared with the primary results table: the range of standard errors increased slightly from 0.069-0.150 to 0.073-0.158. All parameters remain statistically significant. For the engagement slope, the p-value changed from .022 to .034, with no changes to the direction, magnitude, or overall significance of the conclusion.

Table S2. Path coefficients under cluster-robust standard errors (Sensitivity Analysis).

Paths	Unstandardized Coefficient (*SE*)	β	95% C.I.	*p*-value
ab_intercept → ab_slope	0.062 (0.020)	0.28	[0.023, 0.101]	0.002
le_intercept → le_slope	0.027 (0.034)	0.08	[−0.040, 0.094]	0.428
ab_intercept → le_intercept	0.197 (0.060)	0.26	[0.079, 0.315]	< 0.001
ab_intercept → le_slope	0.064 (0.026)	0.25	[0.013, 0.115]	0.014
ab_slope → le_slope	0.346 (0.105)	0.3	[0.140, 0.552]	< 0.001

Compared with the results in the primary table, all four statistically significant paths (original p-values: 0.001 / <0.001 / <0.01 / <0.001) remain significant after adjustment (adjusted p-values: 0.002 / <0.001 / 0.014 / <0.001). The originally non-significant path from le_intercept to le_slope retains non-significance (p = .428). Overall changes in p-values fall within an acceptable range.
